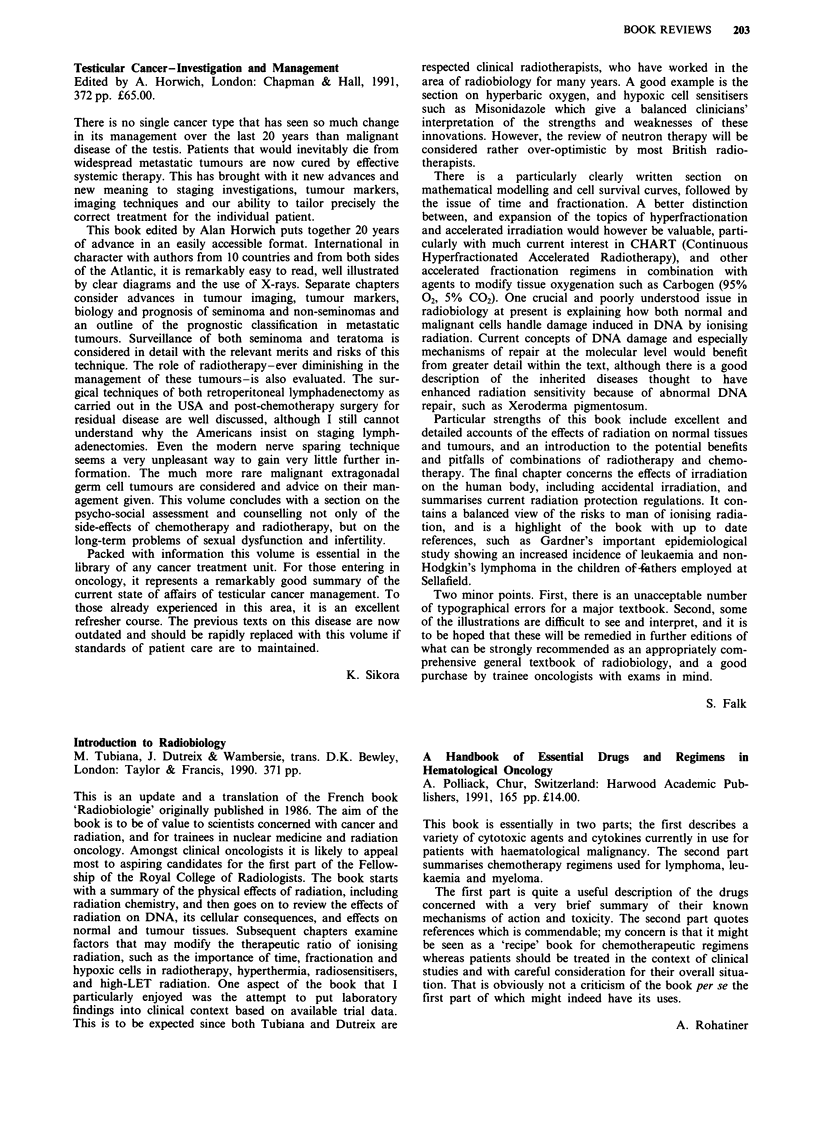# A Handbook of Essential Drugs and Regimens in Hematological Oncology

**Published:** 1993-01

**Authors:** A. Rohatiner


**A Handbook of Essential Drugs and Regimens in Hematological Oncology**
A. Polliack, Chur, Switzerland: Harwood Academic Publishers, 1991, 165 pp. £14.00.

This book is essentially in two parts; the first describes a variety of cytotoxic agents and cytokines currently in use for patients with haematological malignancy. The second part summarises chemotherapy regimens used for lymphoma, leukaemia and myeloma.

The first part is quite a useful description of the drugs concerned with a very brief summary of their known mechanisms of action and toxicity. The second part quotes references which is commendable; my concern is that it might be seen as a 'recipe' book for chemotherapeutic regimens whereas patients should be treated in the context of clinical studies and with careful consideration for their overall situation. That is obviously not a criticism of the book *per se* the first part of which might indeed have its uses.

A. Rohatiner